# Short-Term New Zealand ‘Blackadder’ Blackcurrant Juice Supplementation Improves Learning and Memory in Young Adult Rats

**DOI:** 10.3390/ijms262311568

**Published:** 2025-11-28

**Authors:** Dominic Lomiwes, Alexander P. Kanon, Birgit Ha, Janine M. Cooney, Andrew Scholey, Kirsty A. Lyall, Dwayne J. Jensen, Roger D. Hurst

**Affiliations:** 1 New Zealand Institute for Bioeconomy Science Limited, Private Bag 11030, Palmerston North 4410, New Zealand; alex.kanon@qut.edu.au (A.P.K.); roger.hurst@ag-emissions.nz (R.D.H.); 2 New Zealand Institute for Bioeconomy Science Limited, MARC, Auckland 1025, New Zealand; birgit.ha@plantandfood.co.nz; 3 New Zealand Institute for Bioeconomy Science Limited, Hamilton 3214, New Zealand; janine.cooney@plantandfood.co.nz (J.M.C.); kirsty.lyall@plantandfood.co.nz (K.A.L.); dwayne.Jensen@plantandfood.co.nz (D.J.J.); 4 School of Psychology, University of Northumbria, Newcastle upon Tyne NE1 8ST, UK; andrew.scholey@northumbria.ac.uk

**Keywords:** blackcurrant, polyphenol, learning, memory, stress, antioxidant, corticosterone

## Abstract

Berryfruit consumption has been shown to improve aspects of cognition in humans, and may protect against age-related cognitive decline via antioxidant mechanisms. This study investigated whether short-term supplementation with New Zealand ‘Blackadder’ blackcurrant (BC) juice supports spatial learning and memory in healthy young adult Sprague Dawley rats. Male rats (*n* = 24) received BC juice (*n* = 12; 5.4 mg/kg polyphenols) or a sugar-matched placebo (*n* = 12) prior to each daily trial during a four-day Morris Water Maze (MWM) acquisition phase and a probe trial on day five. BC supplementation significantly reduced cumulative distance (*p* = 0.020) and latency (*p* = 0.030) to the platform. In the probe trial, the trends showed that BC-supplemented rats spent more time in the platform zone. These improvements corresponded with detectable plasma anthocyanins, and trends toward higher hippocampal superoxide dismutase activity (*p* = 0.028, *p*_a_ = 0.140) and lower circulating corticosterone (*p* = 0.052, *p*_a_ = 0.228) in the BC group. These results suggest that BC phytochemicals may support spatial learning and memory. Mechanisms of effect may occur through specific antioxidant-mediated neuroprotective pathways and/or modulation of aspects of the hypothalamic–pituitary–adrenal axis.

## 1. Introduction

Stress is widely recognised as a modulator of learning and memory, with its effects dependent on several factors, including intensity, duration and the specific cognitive processes involved. Preclinical studies have demonstrated an inverted U-shaped relationship between stress and learning and memory, where moderate acute stress can be beneficial while high levels of stress impair performance [1]. These findings are confirmed by clinical studies that show improved memory encoding and consolidation in healthy adults when stress is appropriately regulated and timed [2]. However, high levels of both acute and chronic stress impair memory retrieval and disrupt the integration of new information into existing memory networks [2].

Stress exerts widespread neurobiological effects through the activation of the hypothalamic–pituitary–adrenal (HPA) axis, leading to increased glucocorticoid release. Acute increases in glucocorticoids impair hippocampal synaptic plasticity [3], a mechanism that underlies learning and memory acquisition. Glucocorticoids also increase mitochondrial respiration, shifting the redox balance through reactive oxygen species (ROS) accumulation [4,5], resulting in oxidative stress and associated cellular damage. The hippocampus, a brain region essential for spatial learning and memory, is particularly susceptible to ROS-induced cellular damage, with elevations in ROS associated with deficits in learning and memory, particularly during ageing [6]. Stress also increases dopaminergic and noradrenergic turnover in the brain, which can impair cognitive flexibility under demanding conditions [7,8].

Higher consumption of fruits and vegetables is associated with improved cognitive performance and reduced risk of cognitive decline [9,10]. Contributing to these benefits are flavonoids, a diverse group of polyphenolic compounds, which have attracted considerable attention for their neuroprotective properties. The consumption of flavonoid-rich dietary interventions has been associated with improvements in memory, executive function and processing speed [10,11,12]. These benefits have been attributed to the potent antioxidant and immunomodulatory properties of these compounds, which are proposed to limit the ROS-induced neuronal damage [13] and neuroinflammation [14] that are upregulated during stress. Dietary flavonoids are also bioavailable and detectable in plasma, jejunum, kidney and liver [15,16]. Importantly, they cross the blood–brain barrier (BBB) and localise in cognitive centres like the hippocampus [16,17] to directly influence neuroprotective and cognitive pathways in these brain regions.

Berryfruit are among the richest dietary sources of flavonoids. Preclinical studies have demonstrated that blueberry supplementation ameliorated age- [18,19] and Alzheimer’s disease-related [20] declines in spatial learning and memory in rodents. This is further corroborated by clinical studies reporting improvements in memory performance among older adults [21,22] and healthy children [23] following blueberry supplementation. In addition to their antioxidant properties, improvements in cognitive outcomes from dietary flavonoids in these studies may be partly attributed to their ability to enhance synaptic plasticity through the upregulation of brain-derived neurotrophic factor (BDNF) signalling [24,25].

Recent evidence showed that blackcurrant (BC) supplementation improved scopolamine-induced memory deficits in rodents by mitigating both increased acetylcholinesterase and elevated oxidative stress via antioxidant mechanisms [26]. Previous work by our group and others has reported the efficacy of acute BC supplementation in sustaining attention and mood in healthy adults, particularly during mental fatigue and sub-maximal exercise [27,28]. These effects have been linked to the inhibition of monoamine oxidase (MAO)-B enzyme activity, which may maintain levels of central dopamine, a neurotransmitter associated with attention, learning and memory. We recently identified sarmentosin, a γ-nitrile glycoside present in BC, as a potent MAO-B inhibitor, providing a potential mechanistic explanation for BC’s neuroactive effects [29].

Although other berryfruit have been shown to improve learning and memory, the efficacy of BC in supporting these specific outcomes under stress in healthy individuals without cognitive impairments remains unclear. In this study, we employed the Morris Water Maze (MWM), a widely used stress-inducing behavioural test [30,31], to investigate the benefits of short-term New Zealand ‘Blackadder’ BC juice supplementation on spatial learning and memory in healthy young adult rats. We hypothesised that BC supplementation would increase brain antioxidant capacity to mitigate stress-induced deficits in spatial learning and memory during the MWM task.

## 2. Results

### 2.1. Blackcurrant Anthocyanins Are Rapidly Absorbed After Consumption

The total blackcurrant anthocyanin dose consumed by BC-supplemented rats in this study was 5.4 mg/kg (Table 1). Scaled to a 60 kg human adult, the anthocyanin dose in this study equates to 324 mg in total, which is within the dose range (205–483 mg) used in human clinical trials demonstrating the cognitive benefits of BC supplementation [28,32].

The plasma anthocyanin concentration was determined for each rat from blood samples collected immediately after completion of the probe trial. The administration of the study interventions was timed to coincide with published peak anthocyanin bioavailability during acquisition and probe trials [15]. The absorption of intact BC anthocyanins immediately after the probe trial, which was conducted 30 min post-treatment gavage, was confirmed in the BC treatment group. Compared with previous bioavailability rat studies, the total plasma anthocyanins in the BC group (21.8 ± 1.9 nM) were within the expected concentration range for the anthocyanin dose administered (Figure S1).

### 2.2. Blackcurrant Supplementation Supports Early Learning and Spatial Memory Performance

**Figure 1:**
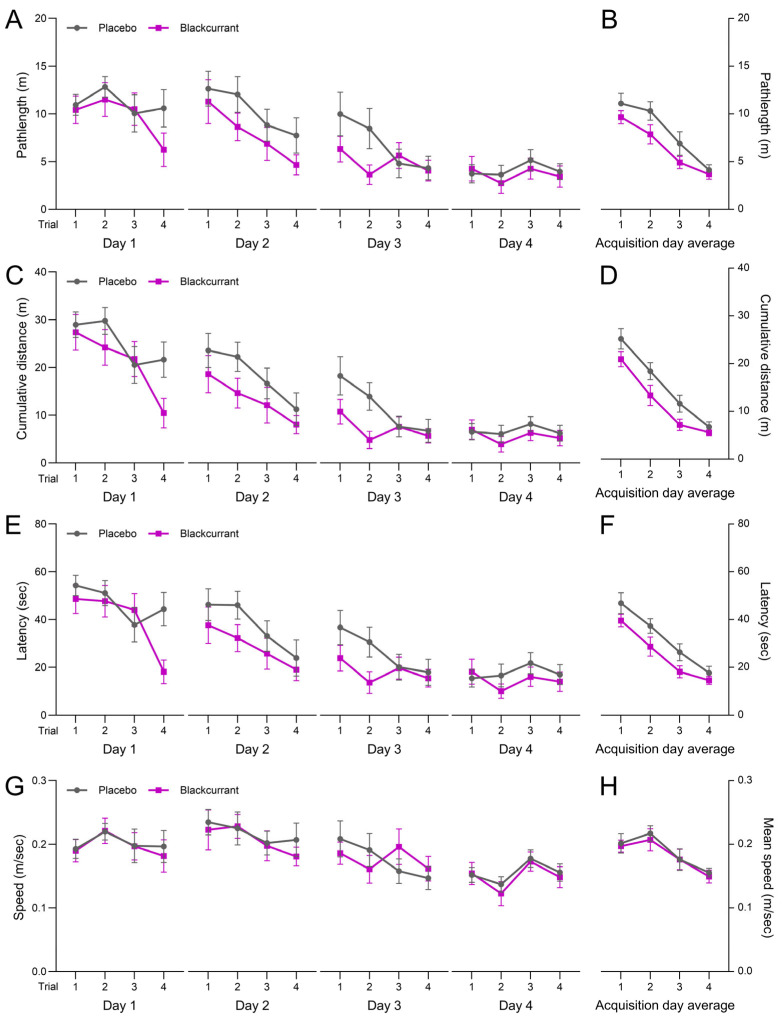
The effects of ‘Blackadder’ blackcurrant (BC) juice or placebo consumption on measures of spatial learning over four acquisition days. Pathlength travelled to the platform (**A**), cumulative distance from the platform (**C**), latency to the platform (**E**) and swim speed while seeking the platform (**G**) were measured during each trial. Values across trials for each acquisition day were averaged to determine the mean pathlength (**B**), cumulative distance (**D**), latency to the platform (**F**) and speed (**H**) over the four-day acquisition period in both BC and placebo groups. Values are means ± SEM.

Pathlength, cumulative distance and latency to the platform across all four acquisition trials over the four trial days (Figure 1A, Figure 1C and Figure 1E, respectively) showed significant main effects for the acquisition trial (*p* < 0.001) and trial day (*p* < 0.001). All three measures progressively decreased with successive trials and days, indicating consistent improvements in spatial learning over the four trial days. A significant main effect for treatment was measured for cumulative distance and latency to the platform (*p* = 0.020 and 0.030, respectively), with mean measures for these outcomes significantly lower in the BC group compared with the placebo. Additionally, a trend toward a significant treatment effect (*p* = 0.077) was observed for the pathlength to the platform, with a trend for lower swimming distances by rats in the BC group compared with those in the placebo group. No significant main effects for acquisition trial and treatment were measured for mean speed (Figure 1G; *p* = 0.142 and 0.735, respectively). However, there was a significant main effect for the trial day (*p* < 0.001), further indicating improvements in spatial learning in both treatment groups over the four trial days.

Values from the four trials conducted on each acquisition day were also averaged to give mean scores for each MWM spatial and memory outcome. A significant main effect of acquisition day was found for all MWM outcomes (*p* < 0.001), with progressive improvements observed with each successive acquisition day. Significant treatment effects were measured for cumulative distance (*p* = 0.020; Figure 1D) and latency to the platform (*p* = 0.030; Figure 1F), with lower mean values in the BC group, indicating that the BC-supplemented rats required less distance and time to reach the platform compared with those in the placebo group. A trend toward a treatment effect was observed for pathlength (*p* = 0.070; Figure 1B), while no significant treatment effect was observed for mean speed (*p* = 0.735; Figure 1H). 

No significant treatment × trial day interactions were observed for any MWM learning outcomes, including latency (*p* = 0.743), cumulative distance (*p* = 0.516), pathlength (*p* = 0.556) or mean speed (*p* = 0.967).

### 2.3. Blackcurrant Supplementation Supports Spatial Memory Retention

**Figure 2:**
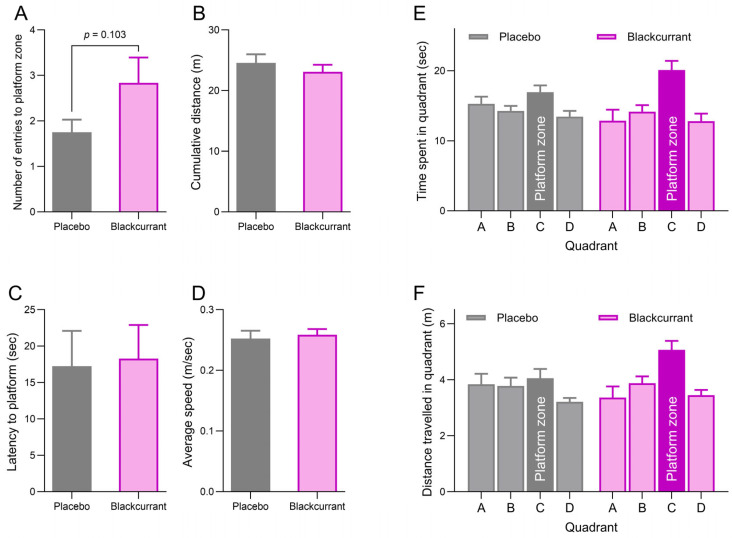
The effects of ‘Blackadder’ blackcurrant (BC) juice or placebo consumption on spatial memory during the probe trial day. The number of entries into the platform zone (**A**), cumulative distance from the platform zone (**B**), latency to enter the platform zone (**C**) and average swim speed (**D**) were measured. Spatial learning was also assessed by measuring the time spent (**E**) and distance travelled (**F**) in each quadrant. Data are presented as means ± SEM.

Spatial memory was tested during the probe trial, where the platform was removed from the pool. A trend toward a treatment effect was observed for the number of entries to the platform zone, with rats in the BC group tending to make more entries than those in the placebo group (Figure 2A; *p* = 0.103). No significant treatment differences were observed for cumulative distance from the platform zone (Figure 2B; *p* = 0.429), latency to the platform zone (Figure 2C; *p* = 0.877) or average speed (Figure 2D; *p* = 0.695).

Although no significant treatment effects were observed for either the time spent (Figure 2E) or distance travelled (Figure 2F) in each quadrant (*p* = 0.998 and 0.369, respectively), both measures showed a significant main effect of quadrant (*p* < 0.001). Post hoc analysis revealed that animals spent more time and swam longer distances in Quadrant C where the platform was previously located, compared with Quadrants A, B and D. Trends toward significant treatment × quadrant interactions for the time spent and distance travelled in quadrant were observed (*p* = 0.080 and 0.083, respectively). These effects correspond with trends of rats in the BC group spending more time and covering greater distances in Quadrant C than in the other quadrants. This pattern was less evident in the Placebo group.

### 2.4. Blackcurrant Supplementation Reduced Plasma Corticosterone, but Had No Effect on Plasma Total Antioxidant Capacity

**Figure 3:**
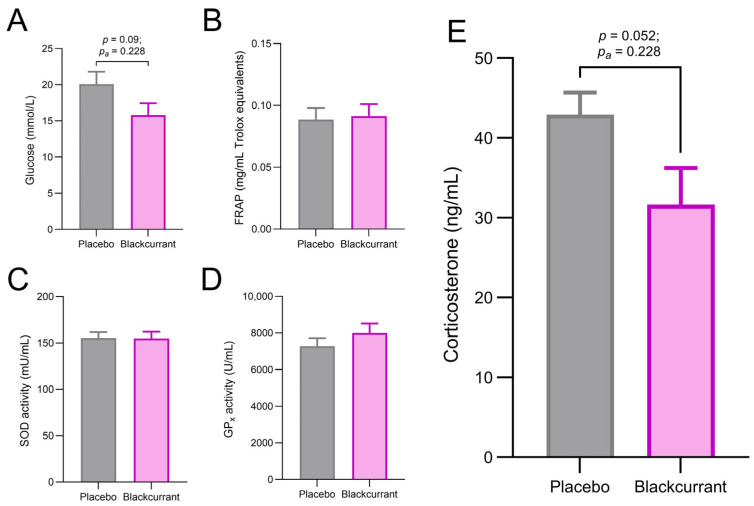
Effects of ‘Blackadder’ blackcurrant (BC) juice or placebo consumption on plasma biomarkers of antioxidant activity and physiological stress after Morris Water Maze (MWM) acquisition and probe trial days. Blood glucose concentrations (**A**), ferric reducing ability of plasma (FRAP) (**B**), erythrocyte superoxide dismutase (SOD) activity (**C**), erythrocyte glutathione peroxidase (GPx) activity (**D**) and plasma corticosterone concentrations (**E**) were measured in blood samples collected immediately following the probe trial. Data are presented as means ± SEM.

A trend toward a treatment effect was observed for blood glucose, with lower concentrations in the BC group compared with the placebo. (Figure 3A; *p* = 0.091, *p*_a_ = 0.228). No significant differences between treatment groups were detected for plasma FRAP (Figure 3B; *p* = 0.835, *p*_a_ = 0.835), erythrocyte SOD activity (Figure 3C; *p* = 0.817, *p*_a_ = 0.835) or GPx activity (Figure 3D; *p* = 0.560, *p*_a_ = 0.835).

A near-significant treatment effect was observed for plasma corticosterone concentrations following the four acquisition days and probe trial. Lower concentrations of corticosterone were measured in the BC group compared with the placebo group (Figure 3E; *p* = 0.052, *p*_a_ = 0.228). While this effect was not statistically significant after FDR adjustment, the unadjusted *p*-value indicates a noteworthy trend that BC supplementation may help attenuate MWM-induced increases in corticosterone.

### 2.5. Blackcurrant Supplementation Enhanced Hippocampal SOD Activity Without Altering Hippocampal Antioxidant Protein Expression

**Figure 4:**
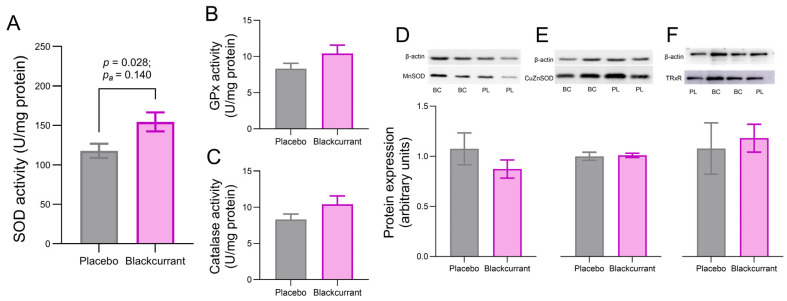
Effects of ‘Blackadder’ blackcurrant (BC) juice or placebo supplementation on hippocampal antioxidant enzyme activity and protein expression following Morris Water Maze (MWM) acquisition and probe trial. Activities of superoxide dismutase (SOD) (**A**), glutathione peroxidase (GPx) (**B**) and catalase (**C**) were measured in hippocampal tissue collected from rats euthanised immediately after the probe trial. Representative Western blots measuring the protein expression of manganese SOD (MnSOD) (**D**), copper-zinc SOD (CuZnSOD) (**E**) and thioredoxin reductase (TRxR) (**F**) in the hippocampus from BC- and placebo-treated rats are shown. Data are presented as mean ± SEM.

The antioxidant capacity of the hippocampus, a brain region critical for learning and memory, was characterised to determine whether improvements in MWM performance from BC supplementation can be attributed to increased hippocampal antioxidant capacity in this group. Hippocampal SOD activity was higher in BC-supplemented rats compared with those in the placebo group (Figure 4A; *p* = 0.028, *p*_a_ = 0.140). Although this effect was no longer significant after FDR adjustment, the unadjusted *p*-value indicates a noteworthy trend that suggests the efficacy of BC supplementation in increasing hippocampal SOD activity. In contrast, no significant treatment effects were observed for the hippocampus GPx (Figure 4B; *p* = 0.155, *p*_a_ = 0.258) and catalase activities (Figure 4C; *p* = 0.143, *p*_a_ = 0.258). Moreover, BC supplementation had no significant effect on the hippocampal expression of antioxidant enzymes MnSOD (Figure 4D; *p* = 0.294, *p*_a_ = 0.534), CuZnSOD (Figure 4E; *p* = 0.822, *p*_a_ = 0.835) or TrxR (Figure 4F; *p* = 0.734, *p*_a_ = 0.835).

### 2.6. Blackcurrant Does Not Affect Hippocampal Mitochondrial Number, BDNF or MAO Activity

**Figure 5:**
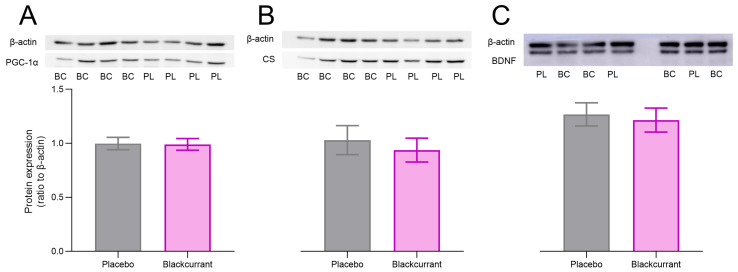
Effects of ‘Blackadder’ blackcurrant (BC) juice or placebo consumption on hippocampal protein expression following Morris Water Maze (MWM) acquisition and probe trial. Protein levels of peroxisome proliferator-activated receptor gamma coactivator 1-alpha (PGC-1α) (**A**), citrate synthase (CS) (**B**) and brain-derived neurotrophic factor (BDNF) (**C**) were measured in the hippocampi of animals collected after the probe trial. Representative Western blots for each protein from BC- and placebo-treated rats are shown. Data are presented as mean ± SEM.

Western blot analysis revealed no significant differences in the expression of hippocampal mitochondrial proteins PGC-1α (Figure 5A; *p* = 0.539, *p*_a_ = 0.835) and CS (Figure 5B; *p* = 0.606, *p*_a_ = 0.835) between treatment groups. Similarly, no significant treatment effect was observed for hippocampal BDNF levels (Figure 5C; *p* = 0.739, *p*_a_ = 0.835). No treatment differences between BC and placebo groups following five days of treatment consumption and MWM training were measured for both MAO-A (*p* = 0.866, *p*_a_ = 0.866) and MAO-B (*p* = 0.459, *p*_a_ = 0.574; Figure S2).

## 3. Discussion

Results reported in this study demonstrate that short-term supplementation with a physiologically relevant dose of New Zealand ‘Blackadder’ BC juice supports spatial learning and memory in healthy young adult rats. These cognitive improvements were accompanied by detectable plasma anthocyanin bioavailability, with notable trends in increased hippocampal SOD activity and reduced plasma corticosterone concentration. Collectively, these findings suggest that BC phytochemicals may support cognitive performance under stress via antioxidant-mediated neuroprotection and modulation of HPA axis activity. The BC dose used in this study was within a range translatable to human consumption, underscoring the potential relevance of these findings for dietary strategies aimed at supporting cognitive function.

The MWM is a well-established paradigm for assessing hippocampal-dependent spatial learning and memory in rodents [33,34]. While many parameters measured from the MWM task can be related to cognitive performance, latency to the platform is considered the most relevant parameter for assessing cognitive effects, as it reflects learning and memory performance. In the present study, BC-supplemented rats outperformed the placebo-fed group across multiple MWM learning outcomes, with the most pronounced improvements occurring during the early acquisition phase. These early improvements may reflect enhanced memory encoding or more efficient spatial navigation strategies, both of which are dependent on hippocampal function. During the probe trial, BC-supplemented rats demonstrated trends toward better spatial memory, spending more time in the platform quadrant, indicative of more accurate recall of the platform location. These findings are consistent with reports that polyphenol-rich interventions, including green tea catechins [35], Ginko biloba [36,37] and (-) epicatechin [38], can improve spatial memory in healthy rodents. In humans, 28-day supplementation of a polyphenol-rich extract of curcumin improved performance on virtual MWM tasks as measured by time spent in the target quadrant [39]. Notably, some studies have reported dose-dependent effects within a bioactive range comparable to the dose used in this study [37,38], underscoring the importance of optimal dosing and that exceeding this range does not necessarily result in greater cognitive benefits [38].

The MWM is inherently stressful for rodents, activating the HPA axis and inducing circulating corticosterone elevations [3,40]. Elevated corticosterone is a well-established modulator of hippocampal function, with sustained increases during learning impairing both learning acquisition and memory. Prolonged corticosterone elevations are associated with poorer MWM performance, including longer latency and pathlength to the platform [31]. Mechanistically, increased corticosterone concentrations can reduce hippocampal glucose uptake, required for neuronal function, impairing memory retrieval [41,42]. Additionally, corticosterone can shift an animal’s strategy from flexible hippocampal-dependent spatial navigation towards more rigid nucleus caudate-based learning [43]. This shift typically results in poorer task performance. In the present study, BC-fed rats showed a trend toward reduced corticosterone, suggesting that BC may attenuate HPA axis activation and help preserve hippocampal engagement under stress.

The neuroprotective potential of BC under stress may also be linked to its antioxidant properties. Stress-induced HPA axis activation is often accompanied by catecholamine release, which increases mitochondrial respiration and ROS production in vulnerable brain regions, including the hippocampus (see review by Spiers et al. [4]). Elevated corticosterone can further exacerbate oxidative stress by suppressing endogenous hippocampal antioxidant defences [44,45]. Given that anthocyanins are known to cross the BBB [16], it is plausible that these compounds directly or indirectly mitigate the stress-induced suppression of antioxidant activity, thereby supporting hippocampal resilience to oxidative stress. In this study, BC-supplemented rats exhibited trends toward higher hippocampal SOD activity compared with the placebo, despite no differences in SOD protein expression between groups. This suggests that BC phytochemicals may enhance post-translational enzyme activity rather than altering SOD expression levels. One plausible mechanism may be through polyphenol-mediated upregulation of mitochondrial NAD-dependent deacetylase sirtuin-3 (SIRT3), which post-translationally deacetylates SOD, thereby increasing its activity and reducing ROS accumulation [46]. While this mechanism has previously been reported with polyphenols from green tea [47], resveratrol [48] and pomegranate [49], direct measurements of SIRT3 or deacetylated SOD were not performed in this study. Therefore, this hypothesis remains speculative and requires further investigation. If confirmed, this would highlight the effects of BC in supporting synaptic integrity and plasticity, ultimately contributing to improved spatial learning and memory.

Although the effect of BC on antioxidant and glucocorticoid-related mechanisms offers a plausible explanation for the observed benefits of BC on spatial learning and memory, other pathways may also contribute. Catecholamines, particularly dopamine and norepinephrine, play central roles in modulating hippocampal-dependent learning [50]. In humans, acute BC supplementation has been associated with improved cognitive performance and mood, accompanied by platelet monoamine oxidase-B (MAO-B) inhibition, reduced circulating prolactin and altered plasma neurotransmitter profiles [27,28,32]. Sarmentosin, a nitrile glycoside, has been identified as a potent MAO-B inhibitor in BC [29]. However, in this study, no evidence of hippocampal MAO inhibition was detected in the BC-supplemented rats. This discrepancy is likely due to differences in the physiology of different species and sensitivity to MAO inhibitors, with rodents exhibiting less responsiveness compared with humans [51]. Therefore, it is unlikely that MAO inhibition contributed to the observed cognitive improvements following BC consumption.

While findings from this study contribute to the growing research evidence for BC’s cognitive benefits, some study limitations should be acknowledged. Although the inherently stressful nature of the MWM task is well documented, confirming stress in the present study using additional behavioural indicators or biomarkers beyond corticosterone would have strengthened this assumption. Such measures could also provide mechanistic insights that underpin BC’s efficacy in supporting spatial learning and memory under stress. Furthermore, the supplementation period was relatively short (5 days), and the durability of the observed cognitive benefits and the long-term effects of blackcurrant supplementation are unknown. Long-term studies are needed to determine whether continued intake sustains or enhances the observed cognitive benefits of blackcurrants reported in this study. Finally, this investigation was restricted to healthy adult male rats without overt cognitive impairments. While this design clarifies the impact of BC under healthy conditions, it is unknown whether similar benefits will extend to females or in a population experiencing cognitive decline. Future research should therefore address sex differences and the benefits of BC in mitigating cognitive decline in rodent models of ageing, neurodegeneration or chronic stress, where oxidative stress and HPA axis dysregulation are more pronounced. Finally, this study employed a single dose, and future research should consider a dose–response study to identify the dose that delivers optimal cognitive effects.

## 4. Materials and Methods

### 4.1. Animals

**Figure 6:**
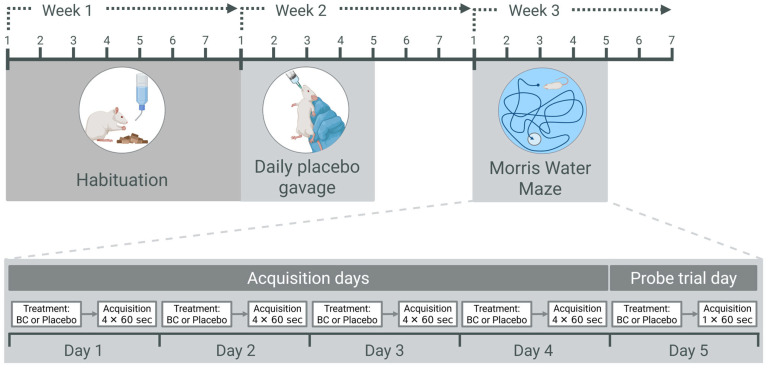
A schematic diagram of the experimental timeline to investigate the effects of ‘Blackadder’ blackcurrant (BC) juice. Following a one-week habituation period, all rats received daily oral gavage of the placebo intervention for five days. On each of four acquisition days in Week 3, animals were gavaged with either BC or placebo prior to completing four Morris Water Maze (MWM) acquisition trials. On Day 5 of Week 3, all rats underwent a single probe trial in the MWM and were immediately euthanised for blood and tissue collection.

Twenty-four three-month-old male Sprague Dawley rats (Harlan Sprague Dawley, Indianapolis, IN, USA) were housed in pairs (matched by weight) in standard stainless steel rat cages with ad libitum access to standard rodent chow and water, maintained on a 12 h reverse day–night rhythm. Following one week of habituation (Week 1) (Figure 6), all rats were handled, weighed and given a daily gavage of placebo intervention by the same individual for five consecutive days (Week 2). This familiarised animals to human contact, oral gavage and to the environment where the MWM was stationed. In the final week of the study (Week 3), rats were randomly assigned to either the ‘Blackadder’ BC juice intervention group (*n* = 12) or placebo (*n* = 12). Rats were administered a single dose of their allocated treatment drink by gavage for five consecutive days, 30 min prior to MWM testing. There was no significant difference in the weight gain between treatment groups during the MWM testing period. All procedures for this study were approved by the AgResearch Group, New Zealand Institute for Bioeconomy Science Limited Animal Ethics Committee (AE Application 12765).

### 4.2. Interventions

Cold-pressed ‘Blackadder’ BC juice from the same batch administered in a previous trial [28] was used in this study. This BC variety is present in commercial formats including EveryDay+ Blackadder Blackcurrant Immunity Syrup (Barker’s of Geraldine, Geraldine, New Zealand) and also marketed as Neuroberry+^®^ by Alphagen NZ Limited (Auckland, New Zealand). The BC juice was stored at −20 °C in 50 mL aliquots and thawed overnight at 4 °C prior to mixing and administering to animals. The phytochemical composition of the BC juice was characterised by high-performance liquid chromatography (HPLC) using the methods described by Schrage et al. [52]. Of the polyphenols that were analysed, delphinidin and cyanidin glycosides comprised 96.7% of the total anthocyanins present in ‘Blackadder’ BC juice (Table 1), which is consistent with the distinct anthocyanin profile of BC [53]. Rats in the BC group received a dose of juice standardised to 5.4 mg total anthocyanins per kilogram of body weight. This dose is within the range of anthocyanin doses used in previous clinical studies investigating the cognitive benefits of blackcurrants [28,32]. The placebo group received a sugar-matched control drink containing glucose, fructose and sucrose in proportions equivalent to those in the BC juice.

### 4.3. Morris Water Maze

The MWM was employed to test each rat’s ability to acquire, remember and use spatial orientation by requiring them to find a submerged platform in a pool using external visual cues [33,54]. A circular black pool with a diameter of 145 cm and filled with water at a depth of 30 cm was used. The water temperature of the pool was maintained at approximately 20 °C. For the purpose of analysis, the pool was divided into four equal quadrants (A, B, C and D) and a hidden black circular platform (10 cm in diameter) was submerged 2 cm below the water surface in quadrant C. The position of the platform remained unaltered throughout the acquisition phase.

To assess spatial learning and memory, rats completed four consecutive days of acquisition training, with each day consisting of four trials, followed by a single probe trial on the fifth day (Figure 6). During every acquisition trial, each animal’s movements were recorded with a digital camera and subsequently analysed using the ANY-maze Video Tracking system (Stoelting Co., Wood Dale, IL, USA). On each trial during the acquisition days, the rats were placed in the water facing the wall in one of the four quadrants. The order of which quadrant the rats were placed in was randomly allocated and varied each trial. A trial lasted until the rat located the platform or for a maximum of 60 s. Once rats located the platform, they were permitted to remain on it for 10 s. If a rat was unable to locate the platform within 60 s, they were gently guided to the platform and left on it for 10 s. Rats were then removed from the platform, dried and returned to their cage to rest for 10 min until the next trial. The total distance, cumulative distance and latency to the platform, and mean swim speed from each trial were recorded and also averaged to provide representative values for each parameter on each acquisition day. On the probe trial day, a single trial was performed (60 s) with the platform removed from the pool. The latency to enter the study platform zone, the number of entries to the platform zone and the cumulative distance from the platform zone were quantified from digital camera footage. Additionally, the time spent and distance travelled in each of the four quadrants during the probe trial were measured.

### 4.4. Blood and Hippocampus Collection and Preparation

Rats were euthanised immediately after their probe trial using pentobarbital. A blood sample was collected by heart puncture and immediately measured for glucose using a handheld glucometer (Accu-Chek^®^, Roche Products Ltd., Auckland, New Zealand). The remaining blood sample was centrifuged to separate erythrocytes and plasma, which were subsequently aliquoted and stored at −80 °C until analysis.

Animals were transcardially perfused with phosphate-buffered saline (PBS), then the brain was removed and the hippocampus was dissected, snap-frozen in liquid nitrogen and stored at −80 °C until analysed. Hippocampus lysates were prepared by homogenising in Sample Buffer (50 mM sodium phosphate, 250 mM sucrose; pH 7.4) with a Bullet Blender™ (Scientific Instruments Services, Inc., Ringoes, NJ, USA) and then centrifuged to remove cell debris. The protein concentrations of hippocampus lysates were measured with a Bio-Rad protein assay kit (Cat # 5000006, Bio-Rad Laboratories, Auckland, New Zealand) according to the manufacturer’s instructions.

### 4.5. Corticosterone Quantification

The plasma corticosterone concentration was quantified using a commercial assay kit (ADI-900-097, Enzo^®^ Life Sciences, Redfern, NSW, Australia) according to the manufacturer’s instructions and calculated as ng/mL plasma. The absorbance of samples and standards at 405 nm was measured with a Fluostar^®^ Optima plate reader (BMG Labtech, Ortenberg, Germany).

### 4.6. Antioxidant Capacity and Enzyme Activities

Ferric reducing ability of plasma (FRAP): Antioxidant capacity was measured using FRAP procedures described by Benzie and Strain [55]. The FRAP of each sample was calculated from a Trolox standard curve, and data were expressed as mg/mL Trolox equivalents.

Glutathione peroxidase (GPx) activity: The GPx activity of erythrocyte cells and hippocampus lysates was quantified with a commercial assay kit (703102, Cayman Chemical Company, Ann Arbor, MI, USA) according to the manufacturer’s instructions. Haemoglobin in erythrocyte samples was measured using Drabkin’s reagent (D5941, Sigma-Aldrich Corp., Auckland, New Zealand) according to manufacturer’s instructions. The GPx activity in erythrocytes and hippocampus lysates was expressed as units/mL haemoglobin and units (U)/mg protein, respectively.

Total superoxide dismutase (SOD) activity: Erythrocytes were lysed in ice-cold deionised water, followed by the addition of 62.5:67.5 ethanol–chloroform solution to the lysate to extract haemoglobin. After centrifugation, the aqueous phase was collected, diluted (1:20) in deionised water and assayed for SOD activity. A volume (100 µL) of erythrocyte lysates or SOD standards (S9697, Sigma-Aldrich Corp.) was pipetted into a clear 96-well plate, and an equal volume of reaction mix (1 mg/mL nitroblue tetrazolium, 30 mg/mL sodium pyrophosphate tetrabasic decahydrate, 1 mg/mL nicotinamide adenine dinucleotide) was added into each well. After 25 µL of 5 µg/mL phenazine methasulphate (PMS) was dispensed into all wells, the absorbance change in each well was measured at 544 nm over 10 min using a plate reader (Fluostar^®^ Omega, BMG Labtech, Ortenberg, Germany). Erythrocyte SOD activity was expressed as mU/mL.

Total SOD activity in hippocampus lysates was measured using methods described by Beauchamp and Fridovich [56] with modifications. Hippocampus lysates or SOD standards (10 µL) were dispensed into a clear 96-well plate, followed by the addition of 190 µL of assay reagent (50 mM Tris-HCl buffer (pH 8.0), 0.1 mM diethylene triamine pentaacetic acid (DTPA), 0.1 mM hypoxanthine, 0.05 mM nitroblue tetrazolium (NBT) and 1.3 mU/mL xanthine oxidase) into all wells. The plate was incubated at room temperature (RT) for 20 min, and the absorbance was measured at 450 nm with a plate reader (Fluostar^®^ Omega, BMG Labtech, Ortenberg, Germany). One unit of SOD activity was defined as the amount needed for 50% dismutation of the superoxide radical, and total SOD activity was expressed as U/mg protein.

Catalase activity: The catalase activity in hippocampus lysates was measured using the Amplex™ Red catalase fluorescent assay kit (A22180, Invitrogen, Thermo Fisher Scientific Ltd., Auckland, New Zealand) according to the manufacturer’s instructions. Fluorescence measurements from the assay were used to calculate enzyme activity, with results expressed as U/mg protein.

### 4.7. Protein Expression of Hippocampal Antioxidant Enzymes, Mitochondrial Proteins and Brain-Derived Neurotrophic Factor

Protein expression of antioxidant enzymes, mitochondrial proteins and BDNF in hippocampus lysates were measured by Western blot. The samples were loaded on 12% SDS polyacrylamide gels, and the proteins were resolved by electrophoresis using a Mini-Protean^®^ vertical electrophoresis system (Bio-Rad Laboratories). Proteins from gels were blotted into polyvinylidene difluoride (PVDF) membranes (Cat # 1620177, Bio-Rad Laboratories) and blocked with 5% non-fat dry milk powder dissolved in TBS-Tween buffer (0.01 M Tris, 0.15 M NaCl, 0.05% Tween-20). Membranes were subsequently probed with primary antibodies diluted in PBS for the protein of interest for 1 h at RT. After washing with TBS-Tween, the membranes were incubated in IgG horseradish peroxidase-conjugated secondary antibody diluted in TBS-Tween for the detection of bound primary antibodies. Membrane-bound primary antibodies were detected with a Clarity™ Western ECL substrate (Cat # 170-5060, Bio-Rad Laboratories) and visualised with a Fujifilm LAS3000 imager (GE Healthcare Life Sciences, Buckinghamshire, UK). For BDNF, membranes probed for BDNF were stripped and then reprobed with antibodies against β-actin to allow for normalisation of protein expression. Details of primary antibodies and their corresponding secondary antibodies used to probe for antioxidant enzymes (manganese (Mn) SOD, copper zinc (CuZn) SOD, thioredoxin reductase (TrxR), mitochondrial proteins (peroxisome proliferator-activated receptor γ co-activator 1 alpha (PGC1-α), citrate synthase (CS) and BDNF) are listed in Supplementary Table S1. Protein expression levels were quantified by densitometric analysis using ImageJ version 1.54f. Band intensities for each target protein were measured and normalised to the corresponding β-actin band within the same lane, which served as a reference protein. This allowed for the relative quantification of protein expression across samples, accounting for lane-specific variation in loading and transfer efficiency.

### 4.8. Hippocampus Monoamine Oxidase Activities

The activity of both MAO-A and -B in hippocampus lysates was measured using an Amplex^®^ Red Monoamine Oxidase assay kit (A12214, Invitrogen, Thermo Fisher Scientific Ltd., Auckland, New Zealand). Briefly, hippocampus lysates were incubated with either p-tyramine (MAO-A and -B substrate) or benzylamine (MAO-B substrate) to facilitate the discrimination of MAO-A and MAO-B activity. Amplex™ Red reagent was then added to the extracts, H_2_O_2_ standards and phosphate-buffered control, and the change in fluorescence (430–560 excitation and 590 nm emission wavelengths) at 37 °C was measured over 10 min using a FLUOstar Omega plate reader (BMG Labtech). Hippocampus MAO-A and -B activities were calculated against H_2_O_2_ standards and expressed as nM H_2_O_2_/µg protein/min.

### 4.9. Plasma Blackcurrant Anthocyanin Bioavailability

Plasma samples (1 mL) were acidified immediately with 5% trifluoroacetic acid (200 µL) and stored at −80 °C until analysis. Prior to the extraction of anthocyanins, samples were further acidified (1:4 6 N HCl:5% formic acid_aq_, 250 µL) and malvidin galactoside (5 ng) was added as an internal standard. Samples were centrifuged (4 °C, 16,000 RCF, 5 min) and proteins removed by precipitation via the addition of acetone (1:4) to an aqueous aliquot (500 µL). The samples were then chilled at −80 °C for 30 min prior to re-centrifuging (16,000 RCF, 4 °C, 5 min) and the acetone was removed via evaporation. Further clean-up to minimise the presence of phospholipids was achieved via liquid–liquid partition of an aqueous aliquot (400 µL) with chloroform. A volume (200 µL) of the aqueous phase was transferred to an autosampler vial for immediate analysis by Liquid Chromatography–Mass Spectrometry (LC-MS).

Anthocyanins were identified by LC-MS using an AB SCIEX QTRAP 5500 (Redwood City, CA, USA) with a Turbo ion spray interface coupled to a Dionex UltiMate 3000 UHPLC system. Compound separation was achieved using an Agilent Poroshell 120 SB-C18 2.1 × 150 mm ID 2.7 µM column (Santa Clara, CA, USA) maintained at 70 °C. Solvents were (A) 5:3:92 acetonitrile/formic acid/water *v*/*v*/*v* and (B) 99.9:0.1 acetonitrile/formic acid *v*/*v* and the flow rate was 600 µL/min. For the initial mobile phase, 100%A was ramped linearly to 90%A at 15 min, then 10%A at 15.1 min and then held for 2 min before resetting to the original conditions. The sample injection volume was 5 µL. MS data was acquired in the positive mode using a multiple reaction monitoring (MRM) method. Anthocyanin concentrations are reported as nM cyanidin glucoside equivalents in plasma.

### 4.10. Statistical Analysis

As no prior data were available on the effects of blackcurrant supplementation on cognitive performance in a MWM paradigm, an adaptive study design was implemented. The study initially began with six animals per treatment group. If significant differences in MWM learning outcomes were observed with this cohort, no additional animals were tested. However, if significance was not achieved, the sample size was increased by six animals per group. Only if results at *n* = 12 per group remained non-significant would the study proceed to the maximum of *n* = 18 animals per group. This adaptive approach was used to minimise animal use while ensuring robust statistical analyses, while remaining consistent with the 3Rs (Replacement, Reduction and Refinement) principles of ethical animal research [57].

Statistical analysis of all data from this study was conducted using Minitab^®^ version 22.2.2. Analysis of data from MWM acquisition and probe trial days was conducted using analysis of variance (ANOVA), based on linear mixed effects models. For MWM acquisition days, fixed effects were set for acquisition trial, acquisition day, treatment and its interactions, and random effects for animal. For the probe trial day, fixed effects were set for treatment, quadrant and its interaction, and random effect for animal. Residuals were checked to confirm that the assumptions of the mixed effects models were met. Specifically, normality and symmetry were assessed using residual Q-Q and histogram plots, and residuals versus fitted value plots were examined to confirm homoscedasticity. Where significance (*p* < 0.05) between main effects or interactions was found, post hoc Tukey analysis was conducted. Treatment differences (BC vs. placebo) for enzyme activity and protein expression were determined by unpaired Student’s *t*-test. As biomarker analyses for antioxidant proteins, mitochondrial proteins, corticosterone, glucose and MAO enzyme activity were secondary outcomes; *p*-values for fixed effects were adjusted using False Discovery Rate (FDR), using the R (version 4.5.2) *p*. adjust command. Both raw *p*-values and FDR-adjusted *p*-values (*p*ₐ) are reported for secondary outcomes. Data are presented as mean ± SEM.

## 5. Conclusions

This study demonstrates that New Zealand ‘Blackadder’ BC juice supports spatial learning and memory in healthy young adult rats. These benefits were accompanied by measurable anthocyanin bioavailability, higher hippocampal SOD activity and a trend toward reduced circulating corticosterone. Together, these findings suggest that blackcurrant constituents may enhance cognitive performance under stress, potentially via SIRT3-mediated deacetylation of SOD, resulting in increased post-translational enzyme activity and reduced ROS accumulation. The results also highlight the translational relevance of the dose used in this study, which aligns with doses used in human trials reporting cognitive benefits. While further research is needed to confirm these effects in both male and female cohorts in humans and to elucidate the precise mechanisms involved, the present study provides compelling pre-clinical evidence supporting blackcurrant as a promising functional food for supporting cognitive performance during physiological stress.

## Figures and Tables

**Table 1:** The polyphenol composition of ‘Blackadder’ blackcurrant juice and the total and anthocyanin dose administered to rats during acquisition and probe trial days in this study.

Compound	Concentration (mg/100 mL)
Caffeoyl quinate	9.1
Caffeic acid glucoside	7.5
p-Coumaroyl quinate	10.2
Epigallocatechin	3.1
Delphinidin glucoside	45.4
Delphinidin rutinoside	178
Cyanidin glucoside	19.5
Cyanidin rutinoside	208
Myricetin rutinoside	13.0
Myricetin glucoside	0
Quercetin rutinoside	4.6
Quercetin glucoside	1.7
Total anthocyanins	466
Total phenolic acids	30.5
Total flavonols	22.3
Total polyphenols	687
Dose consumed	
Anthocyanins (mg)	2.6 ± 0.002
Anthocyanins (mg/kg bodyweight)	5.4 mg/kg
Total polyphenols (mg)	3.9 ± 0.002
Total polyphenols (mg/kg bodyweight)	8 mg/kg

## Data Availability

The data presented in this study are available on request from the corresponding author due to privacy restrictions.

## Supplementary Materials

The following supporting information can be downloaded at: https://www.mdpi.com/article/10.3390/ijms262311568/s1.

## Abbreviations

The following abbreviations are used in this manuscript:

BC	Blackcurrant
MWM	Morris Water Maze
HPA	Hypothalamic–Pituitary–Adrenal
BBB	Blood–Brain Barrier
ROS	Reactive Oxygen Species
BDNF	Brain-Derived Neurotrophic Factor
MAO	Monoamine Oxidase
FRAP	Ferric Reducing Ability of Plasma
GPx	Glutathione Peroxidase
SOD	Superoxide Dismutase
Mn	Manganese
CuZn	Copper Zinc
TrxR	Thioredoxin Reductase
PGC1-α	Peroxisome Proliferator-activated receptor γ Co-activator 1 alpha
CS	Citrate Synthase
LC-MS	Liquid Chromatography-Mass Spectrometry
