# Supplementary material for: Short-Term New Zealand ‘Blackadder’ Blackcurrant Juice Supplementation Improves Learning and Memory in Young Adult Rats
Source: Int J Mol Sci. 2025 Nov 28;26(23):11568. doi: 10.3390/ijms262311568 (PMC12692550; doi:10.3390/ijms262311568)
Supplement: Supplementary file 1 [file ijms-26-11568-s001.zip › ijms-3964690-supplementary.pdf]

Table S1. Details of antibodies used to semi-quantitatively measure the expression of target proteins in the hippocampus tissue of animals in this study.

Antibody	Supplier	Catalogue number
Antioxidant enzyme		
MnZOD	R&D Systems	MAB3419
CuZnSOD	R&D Systems	AF3787
TrxR	R&D Systems	RDSMAB7428S
Mitochondrial and neurotrophic proteins		
PGC1-α	R&D Systems	AF6650
Citrate synthase	Sigma	SAB2701077
BDNF	Santa Cruz Biotechnology	SC-20981
Secondary antibody		
β-actin	Biolegend®	622102

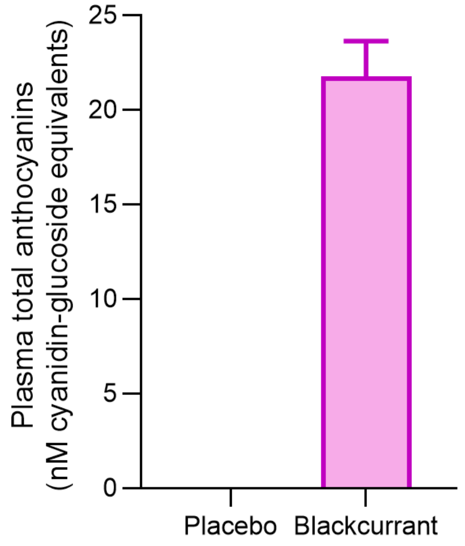

Figure S1. Anthocyanin concentration in plasma 30 min post gavage with "Blackadder" blackcurrant juice or Placebo. Blood samples were collected immediately after completing the probe trial. Data are presented as mean ± SEM.

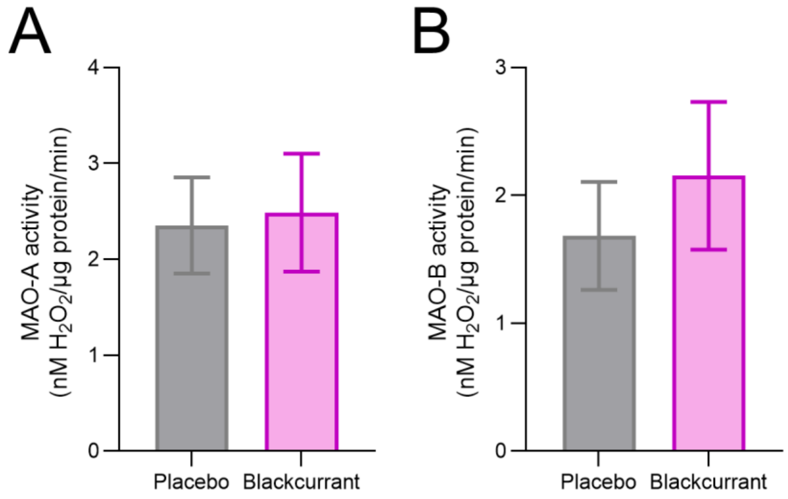

Figure S2. Effects of "Blackadder" blackcurrant (BC) juice or placebo consumption on hippocampal monoamine oxidase (MAO)-A (A) and MAO-B (B) activities following Morris Water Maze (MWM) acquisition and probe trial. Data are presented as mean ± SEM.
